# Robust spatial memory maps encoded by networks with transient connections

**DOI:** 10.1371/journal.pcbi.1006433

**Published:** 2018-09-18

**Authors:** Andrey Babichev, Dmitriy Morozov, Yuri Dabaghian

**Affiliations:** 1 Department of Computational and Applied Mathematics, Rice University, Houston, Texas, United States of America; 2 Lawrence Berkeley National Laboratory, Berkeley, California, United States of America; 3 Berkeley Institute for Data Science, University of California - Berkeley, Berkeley, California, United States of America; 4 Department of Neurology, The University of Texas McGovern Medical School, Houston, Texas, United States of America; King’s College London, UNITED KINGDOM

## Abstract

The spiking activity of principal cells in mammalian hippocampus encodes an internalized neuronal representation of the ambient space—a cognitive map. Once learned, such a map enables the animal to navigate a given environment for a long period. However, the neuronal substrate that produces this map is transient: the synaptic connections in the hippocampus and in the downstream neuronal networks never cease to form and to deteriorate at a rapid rate. How can the brain maintain a robust, reliable representation of space using a network that constantly changes its architecture? We address this question using a computational framework that allows evaluating the effect produced by the decaying connections between simulated hippocampal neurons on the properties of the cognitive map. Using novel Algebraic Topology techniques, we demonstrate that emergence of stable cognitive maps produced by networks with transient architectures is a generic phenomenon. The model also points out that deterioration of the cognitive map caused by weakening or lost connections between neurons may be compensated by simulating the neuronal activity. Lastly, the model explicates the importance of the complementary learning systems for processing spatial information at different levels of spatiotemporal granularity.

## Author summary

The reliability of our memories is nothing short of remarkable. Synaptic connections between neurons appear and disappear at a rapid rate, and the resulting networks constantly change their architecture due to various forms of neural plasticity. How can the brain develop a reliable representation of the world, learn and retain memories despite, or perhaps due to, such complex dynamics? Below we address these questions by modeling mechanisms of spatial learning in the hippocampal network, using novel algebraic topology methods. We demonstrate that although the functional units of the hippocampal network—the place cell assemblies—are unstable structures that may appear and disappear, the spatial memory map produced by a sufficiently large population of such assemblies robustly captures the topological structure of the environment.

## Introduction

Functioning of the biological networks relies on synaptic and structural plasticity processes taking place at various spatiotemporal timescales [1–4]. For example, the so-called place cells in mammalian hippocampus learn to spike within specific locations of a new environment (their respective place fields) in a matter of minutes and then exhibit slow tuning of their firing rates for weeks [5–7]. The functional architecture of the hippocampal network constantly changes due to formation, adaptation and pruning of the synaptic connections via fast and slow plasticity mechanisms [8]. Its key components—the dynamical cell assemblies [9, 10]—may emerge from place cell coactivities and disappear due to reduction or cessation of spiking at working and intermediate memory timescales, between minutes [11, 12] and hundreds of milliseconds [13, 14]. In contrast, spatial memories in rats can last much longer [15, 16], which poses a principal question: how can a rapidly rewiring network produce and sustain a stable cognitive map? In the following, we address this question by modeling a population of dynamical place cell assemblies and study the effect produced by the network’s transience on the large-scale representation of space, using algebraic topology tools. In particular, we demonstrate that despite rapid changes in its synaptic architecture, a transient cell assembly network can encode a stable large-scale topological map within a biologically plausible period.

**Fig 1:**
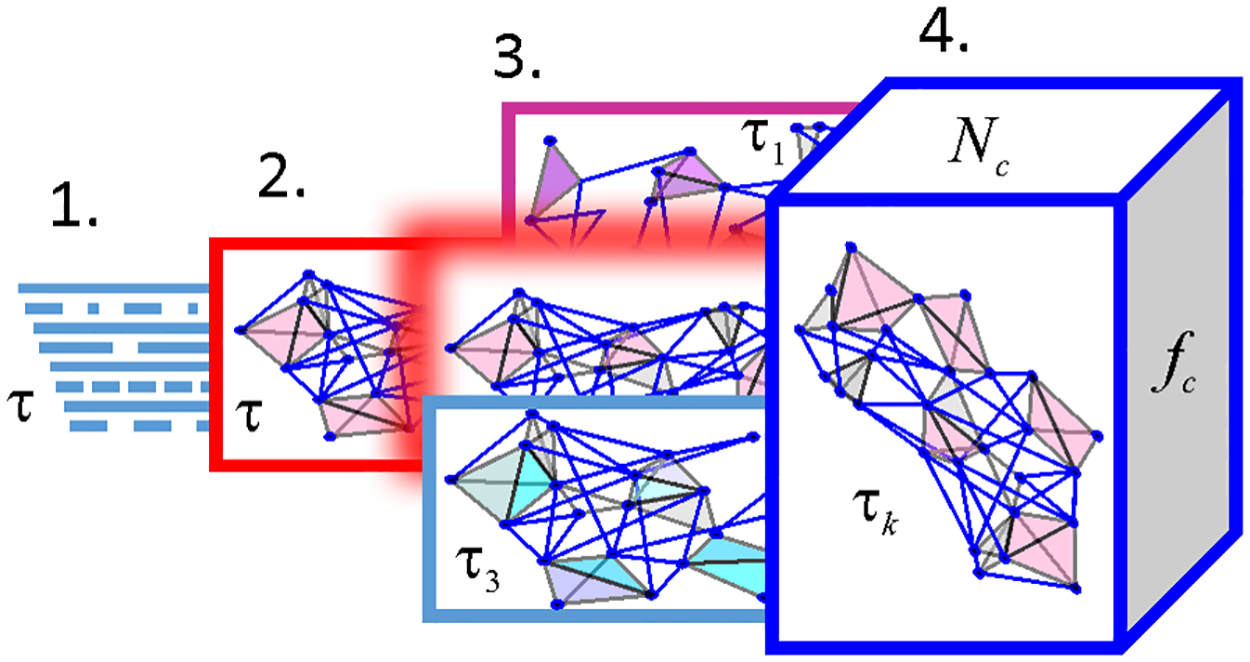
Study outline. 1. Dynamics of transient connections. 2. Dynamics of the transient network represented by a “flickering” simplicial complex. 3. Dependence of the network’s dynamics on the transience rate, *τ*_*k*_, studied in several setups, including “quenched and random complexes. 4. Dependence of the results on the parameters of place cell activity—the mean ensemble firing rate *f*_*c*_, and the number of active cells, *N*_*c*_.

The paper is organized as follows. We start with a general outline of the key ideas behind the topological approach and describe a schematic model of a transient cell assembly network. We then study the statistics of its connections’ turnover, the resulting dynamics of the network as a whole and of the spatial map encoded by this network (Fig 1). The results are tested for several connection decay rates, in different setups and summarized in the Discussion. The required mathematical and computational details are provided in the Methods section.

### The topological model

#### General outline

Our approach is based on recent experimental results [17–19], which suggest that the hippocampal spatial map derived from place cell co-firing emphasizes contiguities between locations and the temporal sequence in which they are experienced. In other words, this map is topological in nature, i.e., akin to a subway map, as opposed to a topographical city map—a property that is also manifested at the cognitive level [20–24]. From the computational perspective, this observation suggests that topological instruments can be used to study the emergence of the hippocampal maps from the place cells’ spiking inputs.

Our model of the hippocampal network is based on a schematic representation of the information supplied by a population of spiking place cells [25–29]. First, a group of coactive place cells, *c*_0_, *c*_1_, …, *c*_*n*_ is represented by an abstract simplex *σ* = [*c*_0_, *c*_1_, …, *c*_*n*_]—a basic object from algebraic topology that may be viewed geometrically as a *n*-dimensional tetrahedron with *n* + 1 vertexes (see Methods). Due to spatial tuning of the place cell activity, each individual coactivity simplex may also be viewed as a representation of the spatial overlap between the corresponding place fields. Together, the full collection of such simplexes forms a simplicial “coactivity” complex T that represents spatial connectivity among the place fields that cover a given environment E, i.e., the structure of the place field map $M_{E}$ (see Methods).

**Fig 2:**
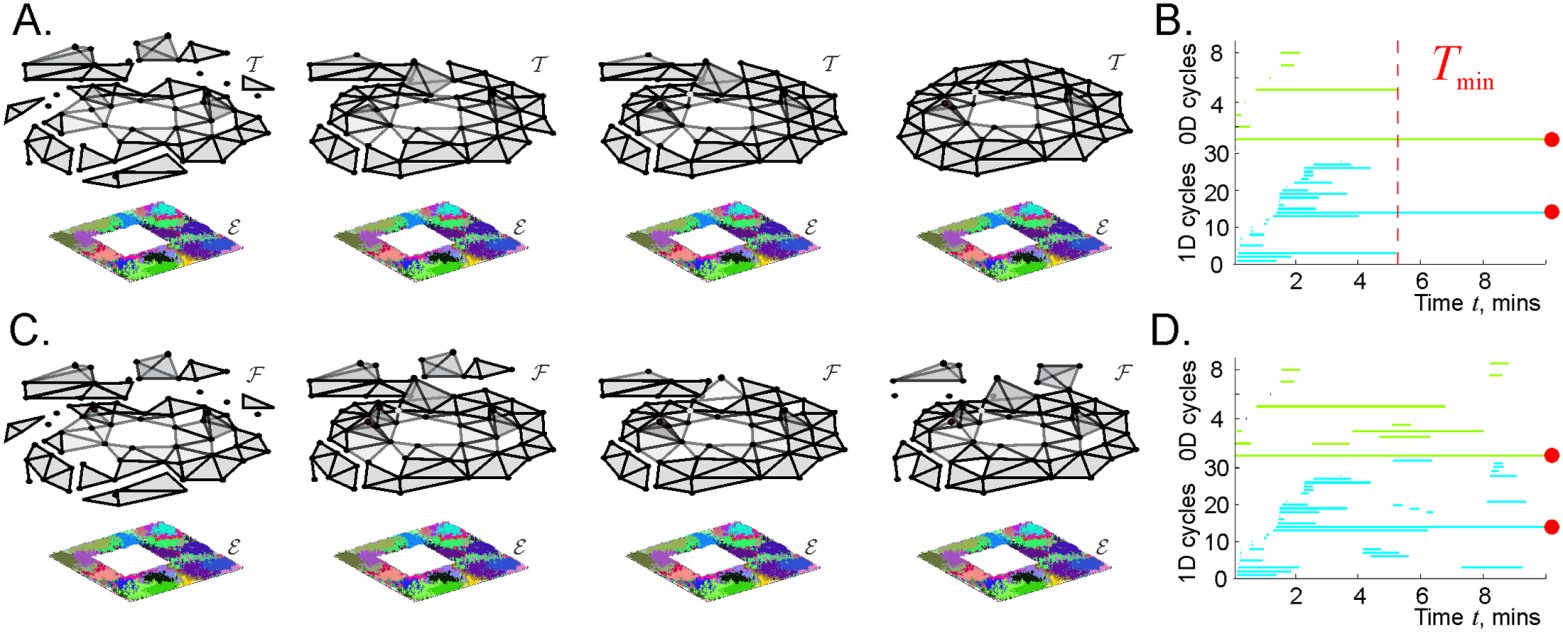
Topological structure of the perennial and decaying coactivity complexes. A: Simulated place field map $M_{E}$ of a small planar environment E with a square hole in the middle (see Methods). Four consecutive snapshots illustrate the temporal dynamics of the coactivity complex: at the early stages of navigation the complex is small and fragmented, but as the topological information accumulates, the transient topological loops disappear, yielding a stable topological shape that is equivalent to the shape of the underlying environment. B: The timelines of topological loops in a steadily growing simplicial complex computed using persistent homology methods: the timelines of disconnected pieces (0*D* loops) are shown by light-blue lines and the timelines of one-dimensional holes (1*D* loops) are light-green. Most loops are spurious, i.e., correspond to accidental, short-lasting structures in $T(M_{E})$. The persistent topological loops (marked by red dots) represent physical features of the environment E, i.e., its main connected component and the central hole. The time *T*_min_ required to eliminate the spurious loops can serve as a theoretical estimate of the minimal time needed to learn path connectivity of the environment. C: If the simplexes may not only appear but also disappear, then the structure of the resulting “flickering” coactivity complex $F(M_{E})$ may never saturate. D: The timelines of the topological loops in such complex may remain interrupted by opening and closing topological gaps produced by decays and reinstatements of its simplexes.

This process of accumulation of the topological information can be represented by the dynamics of the coactivity complex. At the beginning of navigation, the complex $T(M_{E})$ contains a few simplexes that correspond to the few coactive place cell combinations that had time to appear. At this stage, the coactivity complex T is typically split into several disconnected pieces (subcomplexes), riddled with holes. Physiologically, these pieces may be viewed as fragments of the emerging cognitive map (Fig 2A). If the parameters of spiking activity fall within the biological range of values, then, as more instances of coactivity are produced, the coactivity complex $T(M_{E})$ grows and eventually assumes a shape that is topologically equivalent to the shape of the navigated environment.

The topological structure of a steadily growing coactivity complex can be described using persistent homology theory methods [30, 31]. In particular, this theory allows detecting topological loops in $T(M_{E})$—closed chains of simplexes identified up to topological equivalence [32]—on a moment-by-moment basis (Fig 2B). Such loops provide a convenient semantics for describing how $T(M_{E})$ unfolds in time. For example, the number of inequivalent topological loops that can be contracted to a zero-dimensional vertex defines the number of the connected components in $T(M_{E})$; the number of loops that contract to a one-dimensional chain of links defines the number of holes and so forth. In mathematical literature, the number of *k*-dimensional topological loops in a space *X* is referred to as its *k*-th Betti number, *b*_*k*_(*X*), and the list of all Betti numbers defines the topological barcode, $b\left(X\right)=(b_{0}\left(X\right),b_{1}\left(X\right),\ldots )$ of *X* [33]. For example, the simply connected, square environment E with a single hole in the middle (Fig 2A and Methods) has the Betti numbers $b_{0}\left(E\right)=b_{1}\left(E\right)=1$, with no higher order loops, $b_{k>1}\left(E\right)=0$; hence its topological barcode is b(E)=(1,1,0,0,…). The time *T*_min_ required by the coactivity complex to produce the topological barcode of the underlying environment, b(T)=b(E), can serve as a theoretical estimate for the “learning” period needed to accumulate spiking data for establishing the large-scale spatial structure of the environment [25–29].

The construction of the coactivity complexes may be adopted to reflect physiological aspects of the hippocampal network. For example, the simplexes of the coactivity complex may represent not just arbitrary combinations of coactive cells, but the neuronal assemblies—groups of cells that jointly elicit spiking activity in the downstream neurons. As mentioned in the Introduction, these assemblies are unstable, transient structures that are recycled, according to different estimates, at the timescale between minutes to hundreds of milliseconds [9, 10].

In order to represent this transience, the simplexes of the coactivity complex are allowed to appear and to disappear, i.e., “flicker,” following the appearances and disappearances of the corresponding cell assemblies. As a result, certain parts of the resulting “flickering” coactivity complex $F(M_{E})$ complex may produce holes, fractures or fragment into pieces that can inflate or shrink, at different rates and in various sequences (Fig 2C). The topology of such a complex cannot, in general, be described using ordinary persistent homology theory methods (Fig 2D), and requires a different mathematical apparatus—Zigzag persistent homology theory, outlined in the Methods section and in [34–36].

#### Implementation

An efficient implementation of the coactivity complex is based on a classical “cognitive graph” model of the hippocampal network [37–40]. In this model, each active place cell *c*_*i*_ corresponds to a vertex *v*_*i*_ of a graph G, and the connections between pairs of cells (physiological or functional) are represented by the links *ς*_*ij*_ = [*v_i_*, *v_j_*] of G. The assemblies of place cells *c*_1_, *c*_2_, …, *c*_*n*_ (“synaptically interconnected networks” in terminology of [10]) can then be naturally interpreted as fully interconnected subgraphs between the corresponding vertexes, i.e., as the maximal cliques *ς* = [*v*_1_, *v*_2_, …, *v_n_*] of G [28, 29]. The connection with the topological model described above comes from the observation that cliques, as combinatorial objects, can be viewed as simplexes spanned by the same sets of vertexes. In other words, the collection of cliques of any graph *G* defines the so-called clique complex Σ(*G*) [41], and hence the set of the coactivity cliques of G produces a coactivity complex associated with the cognitive graph. Such a complex effectively accumulates the information about place cell coactivity at various timescales, capturing the correct topology of planar [25–28] and voluminous [29] environments within minutes.

This construction provides a suitable ground for modeling a population of dynamical cell assemblies. Specifically, one can use a coactivity graph with appearing and disappearing (flickering) links to describe the appearing and disappearing connections in the hippocampal network. The topological shape of the corresponding flickering coactivity complex, will then represent the net topological information encoded by this network. This constitutes a simple phenomenological model that connects the information provided by individual dynamical place cell assemblies and their physiological properties (e.g., the rate of their transience) to the structure of the large-scale topological maps encoded by the cell assembly network as a whole. We implemented this model using the following basic assumptions.

*Decay of the connections*. A simple description of a transient network can be given in terms of the probabilities of the links’ appearances and disappearances at a given moment. For the latter, we adopt a basic “decay” model, in which an existing link *ς*_*ij*_ between cells *c*_*i*_ and *c*_*j*_ can disappear with the probability

$$\begin{array}{c} p_{ij}\left(t\right)=\frac{1}{\tau_{ij}}e^{-t/\tau_{ij}}, \end{array}$$

where the time *t* is counted from the moment of the link’s last appearance and the parameter *τ*_*ij*_ defines its mean decay time. The decay times of the higher order cliques in the coactivity graph (i.e., of the higher order cell assemblies in the hippocampal network) are then defined by the corresponding links’ half-lives.

In a physiological cell assembly network, the decay times *τ*_*ij*_ are distributed around a certain mean *τ* with a certain statistical variance [42]. However, in order to simplify the current model and to facilitate the interpretation of its outcomes, we attribute a single value *τ*_*ij*_ = *τ* to all links in G and use a unified distribution

$$\begin{array}{c} p_{0}\left(t\right)=\frac{1}{\tau }e^{-t/\tau }, \end{array}$$ (1)

to describe the deterioration of all the connections within all cell assemblies. Thus, *τ* will be the only parameter that describes the decay of the functional connections in the model. We will therefore use the notations $G_{\tau }$ and $F_{\tau }$ to refer, respectively, to the flickering coactivity graph with decaying connections and to the resulting flickering clique coactivity complex with decaying simplexes.

*Appearances and rejuvenations of the connections*. A connection *ς*_*ij*_ in the graph G appears if the cells *c*_*i*_ and *c*_*j*_ become active within a *w* = 1/4 second period (biologically, this corresponds to two consecutive periods of the *θ*-rhythm [26, 43]). The subsequent coactivities of the pair [*c*_*i*_, *c*_*j*_] either reinstate the link *ς*_*ij*_ (if it has disappeared by that moment) or rejuvenates it (i.e., its decay restarts). As a result, the links’ actual or *effective* mean lifetime *τ*_*e*_ may differ from the proper decay time *τ* that defines the expected lifetime of an unperturbed connection. Indeed, if the connection *ς*_*ij*_ that appeared at a moment *t*_1_, does not disappear by the moment *t*_2_ when it reactivates, then its net expected lifetime becomes *t*_2_ − *t*_1_ + *τ*. If it does not decay before being “rejuvenated” again at a later moment *t*_3_, then its net expected lifetime is *t*_3_ − *t*_1_ + *τ* and so forth. Notice however, that since place cells’ spiking in learned environments is stable [44], the vertexes in the coactivity complex $F_{\tau }$ appear with the first activation of the corresponding place cells and then never disappear.

*Fixed geometric parameters*. The series of instances at which a given combination of cells may become active is defined by the geometry of the place field map $M_{E}$ and by the times of the rat’s visits into the locations where the corresponding place fields overlap [45, 46]. In order to study how the dynamics of the Betti numbers $b_{k}\left(F_{\tau }\left(M_{E}\right)\right)$ depends on the links’ decay time *τ*, we selected a specific trajectory *γ*(*t*) and used place field maps $M_{E}$ that induce coactivity complexes with the correct topological shape in the “perennial” (*τ* = ∞) limit [25, 26]. In the following, we will omit references to these parameters in the notations of the coactivity graph or the coactivity complex, and write simply $G_{\tau }$ and $F_{\tau }$.

*Restricted dimensionality*. Although the coactivity complex is multidimensional [28], for a topological description of a planar environment it suffices to consider only the two-dimensional skeleton of $F_{\tau }$, i.e., the collection of second and third order connections (i.e., second or third order cliques of $G_{\tau }$ or two- or three-vertex simplexes of $F_{\tau }$). Thus, in the following we will compute the coactive pairs and triples of the simulated neurons in order to study the topological properties of $F_{\tau }$ as function of *τ* in the lowest two dimensions.

A priori, one would expect that if *τ* is too small, then the flickering complex $F_{\tau }$ deteriorates too rapidly to produce a stable topological representation of the environment. In contrast, if *τ* is too large, then the effect of the decaying connections will not be significant. Thus, our goal will be to identify just how rapidly the coactivity simplexes can recycle while preserving the net topological structure of $F_{\tau }$. Physiologically, this will provide an estimate for how rapidly the hippocampal cell assemblies can rewire without jeopardizing the integrity of the topological map of the environment.

## Results

**Fig 3:**
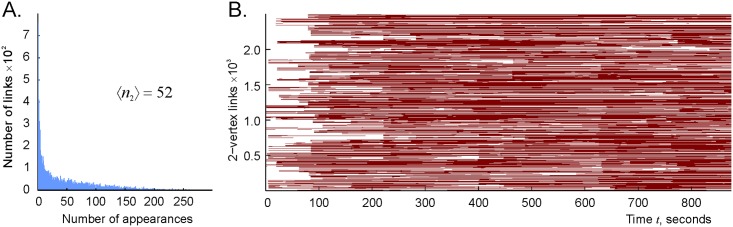
Time course of pairwise coactivity. A: Histogram of the number of times that a given connection *ς*_2_ activates, computed for the place field map illustrated on Fig 2 (for the corresponding occupancy map, see Methods). On average, a connection is activated about 50 times over the 25 min navigation period, i.e., once every 30 secs, although most links activate only a few times whereas some of them may appear hundreds of times. B: Timelines of the links of the coactivity graph G.

To start the simulations, we reasoned that in order for the flickering complex $F_{\tau }$ to accumulate a sufficient number of simplexes and capture the topology of the environment, its simplexes should not disappear between two consecutive coactivities of the corresponding cell groups. In other words, the characteristic lifetime of the links of the coactivity graph should exceed the typical interval between two consecutive activations of the corresponding cell pairs. First, we simulated a map produced by *N* = 300 place cells with mean maximal firing rate *f* = 14 Hz and mean place field size of 20 cm; a typical link *ς* in the corresponding connectivity graph G activates about 〈*n*_2_〉 = 50 times during the *T*_*tot*_ = 25 min navigation period, i.e., the mean activation frequency is *f*_2_ ≈ 1/30 Hz (Fig 3). Hence, in order to make room for the rejuvenation effects, we first tested the decay time of *τ* = 100 secs, which is about three times longer than the inter-activity period and by an order of magnitude smaller than the total navigation time *τ* ≈ *T*_*tot*_/15.

**Fig 4:**
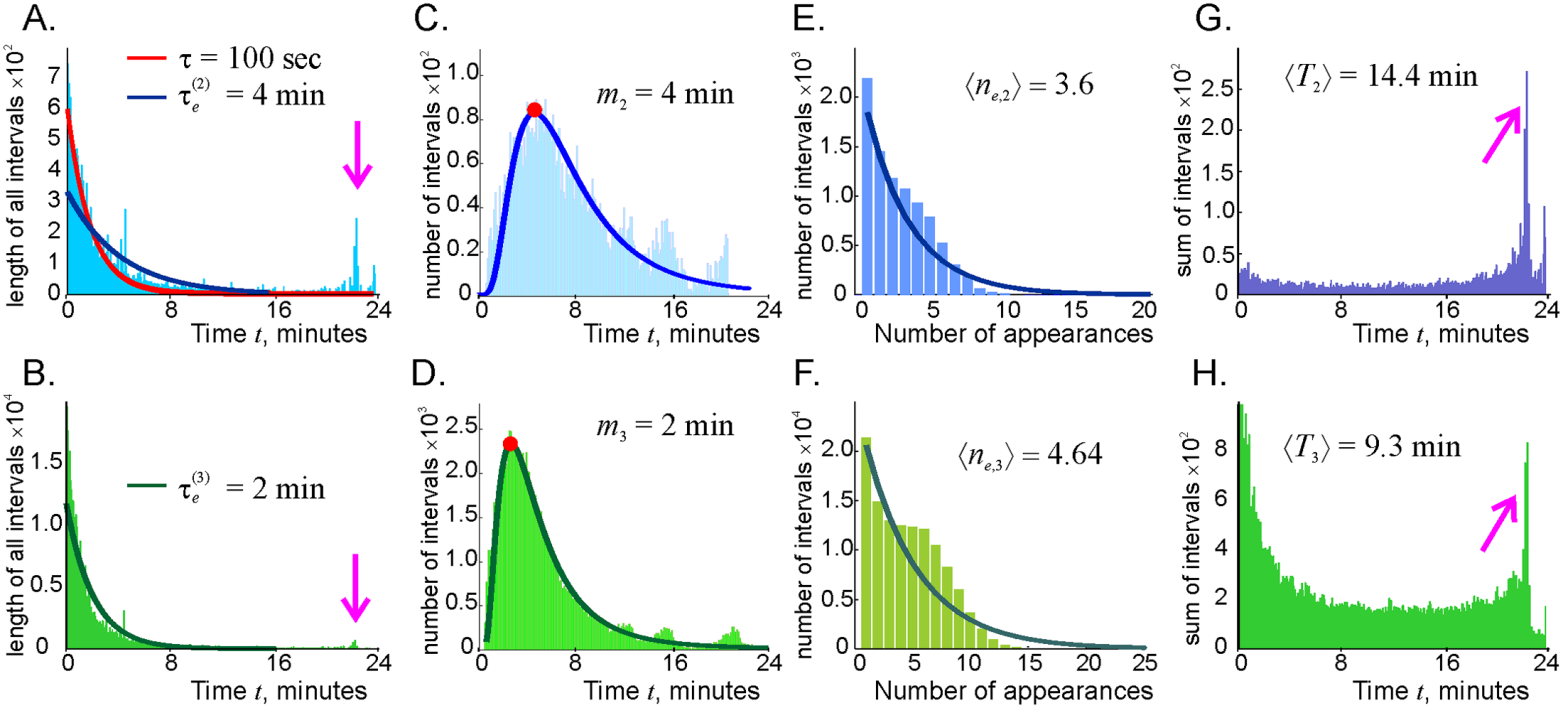
Connection dynamics. A: The histogram of the time intervals between the consecutive births (*b*_*i*_) and deaths (*d*_*i*_) of the links, $\Delta t_{\varsigma_{2},i}=t_{\varsigma_{2}}^{\left(d_{i}\right)}-t_{\varsigma_{2}}^{\left(b_{i}\right)}$, *i* = 1, 2, …. The mean of the exponential that fits the left side of the histogram (dark-blue line) is shown at the top of the panel. The pink arrow points at the population of the “survivor” links. The red line marks the distribution (1). B: Similar histogram for the third order simplexes. The histograms of the lifetimes averaged over all the instances of a given simplexes’ appearances $\Delta t_{\varsigma_{2}}={\langle t_{\varsigma_{2}}^{\left(d_{i}\right)}-t_{\varsigma_{2}}^{\left(b_{i}\right)}\rangle }_{i}$ (panel C) and $\Delta t_{\varsigma_{3}}={\langle t_{\varsigma_{3}}^{\left(d_{i}\right)}-t_{\varsigma_{3}}^{\left(b_{i}\right)}\rangle }_{i}$ (panel D). The modes of the resulting lognormal distributions (solid lines), *m*_2_ and *m*_3_, correspond to the means shown on panels A and B. The histograms of the number of times the link and the triple connections activate during the navigation period are shown on the panels E and F. The exponential fits are shown by solid lines, with the means shown at the top of the panels. The distributions of total existence times for the second (panel G) and third (panel H) order simplexes, with the averages that are approximately equal to the product of the mean effective lifetime and the mean number of appearances, Δ*T*_*e*,*ς*_ ≈ *n_e,ς_τ_e,ς_*.

A histogram of the time intervals $\Delta t_{\varsigma_{i}}$ between the *i*^*th*^ consecutive birth (*b*_*i*_) and death (*d*_*i*_) of a link *ς*, $\Delta t_{\varsigma_{i}}=t_{\varsigma }^{\left(d_{i}\right)}-t_{\varsigma }^{\left(b_{i}\right)}$, shows that the distributions of the connections’ effective lifetimes is bimodal (Fig 4A). The relatively short (Δ*t* ≤ 10*τ*) lifetimes are exponentially distributed, implying that these connections are short-lived (the mode of the exponential distribution vanishes) and may be characterized by the effective decay times that are about twice higher for the links, $\tau_{e}^{\left(2\right)}\approx \tau$, than for the triple connections, $\tau_{e}^{\left(3\right)}\approx \tau$ (Fig 3). In addition, the bulging tails of the distributions shown on Fig 4A and 4B represent an emergent population of long-lived connections, i.e., a set of “survivor” simplexes that persist throughout the navigation period (Δ*t*_*ς*_ ≈ *T_tot_*).

In other words, the net structure of the lifetimes’ statistics suggests that the coactivity complex contains a stable “core” formed by a population of surviving simplexes, enveloped by a population of rapidly recycling, “fluttering,” simplexes. The *mean* lifetime of each individual link, averaged over all the appearances across the entire navigation period, $\Delta t_{\varsigma_{2}}={\langle t_{\varsigma_{2}}^{\left(d_{i}\right)}-t_{\varsigma_{2}}^{\left(b_{i}\right)}\rangle }_{i}$, can be approximated by a lognormal distribution with the mode *m*_2_ ≈ 4 minutes (Fig 4C), which corresponds to the mean lifetime of the “fluttering” connections (Fig 4A). Similarly, a typical third-order simplex appears for about two minutes (Fig 4D), as suggested by the mean of the distribution shown on Fig 4B. Thus, on average, both the coactivity graph $G_{\tau }$ and the corresponding coactivity complex $F_{\tau }$ exhibit persistent structures, despite rapid flickering of the individual connections.

The rejuvenation of simplexes also affects the frequency of their (dis)appearances. As shown on Fig 4E and 4F, a typical link and a typical third order connection disappear about 4 − 5 times during the navigation period, which is by an order of magnitude less than the links’ activation rate (Fig 4B). Thus, a typical simplex rejuvenates about 10 times before getting a chance to decay. The histograms of the net lifetimes, i.e., of the total time that a given link or a clique spends in existence (Δ*T*_*ς*_ = Σ_*i*_Δ*t_ς,i_*) shown on Fig 4G and 4H exhibit an even more salient contribution of the survivor simplexes. Note that the average net lifetime is approximately equal to the product of the mean effective lifetime and the mean number of appearances, Δ*T*_*e*,*ς*_ ≈ *n_e,ς_τ_e,ς_*, as expected.

### Dynamics of the flickering coactivity complexes

**Fig 5:**
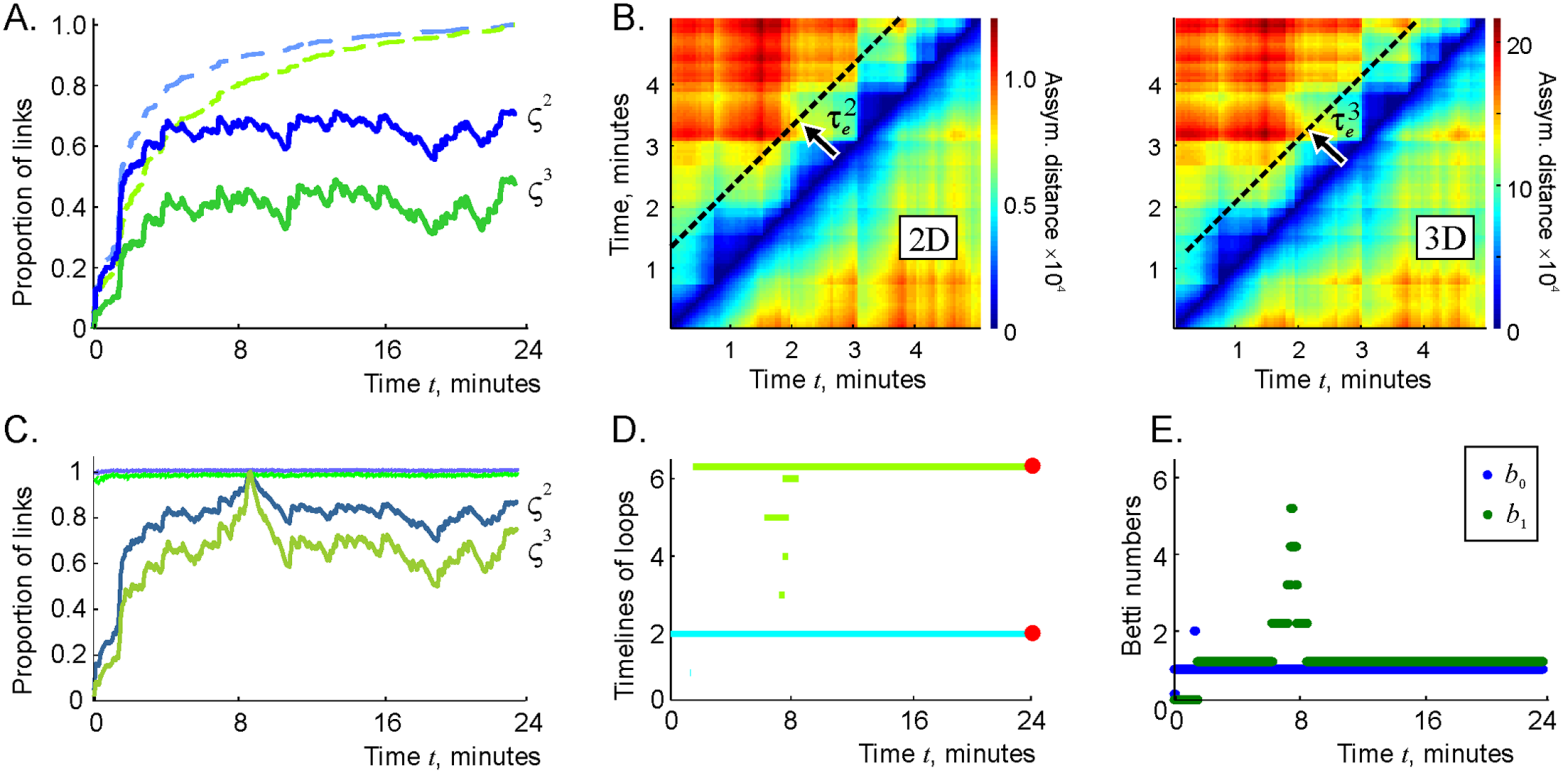
Dynamics of the flickering complex and of its topology. A: The number of links *N*_2_(*t*) in the flickering complex $F_{100}\left(t\right)$ (blue trace) compared to the number of links in the perennial complex $F_{\infty }\left(t\right)$ (dashed light-blue trace). The corresponding numbers of triple connections *N*_3_(*t*) are shown by the green and the dashed light-green traces, respectively. B: The matrix of asymmetric distances $d_{ij}^{\left(2\right)}$ and $d_{ij}^{\left(3\right)}$ (note the difference in scales shown by the color bars), computed over a five minutes time interval. The changes in the coactivity complex accumulate at the *τ*_*e*_ timescale. C: The proportions of second and third order connections shared by the coactivity complexes at the consecutive moments, computed for links (top light-green line) and for the triple connections (top light-blue line) closely follow the 100% mark, which implies that $F_{\tau }\left(t\right)$ deforms slowly. The numbers of connections present at a moment of time when the coactivity complex is inflated (t_*_ ≈ 9 min) that are also present at another moment *t*, $N_{k}\left(F_{\tau }\left(t_{*}\right)\cap F_{\tau }\left(t\right)\right)$, fluctuate around ∼ 80% for links (blue trace) and ∼ 60% for triple connections (green trace), implying that the same set of connections is being reactivated. D: Timelines of 0*D* (light-blue) and 1*D* (light-green) topological loops in $F_{\tau }\left(t\right)$ indicate a splash of topological fluctuations near the inflation time t_*_ = 9 minutes. During other periods, $F_{\tau }\left(t\right)$ contains only one persistent loop in each dimension. E: The instantaneous Betti numbers, $b_{0}\left(F_{\tau }\right)$ and $b_{1}\left(F_{\tau }\right)$ increase around t_*_ = 9 min, but retain their physical values $b_{0}\left(F_{\tau }\right)=b_{1}\left(F_{\tau }\right)=1$ for the rest of the navigation period, which implies that, despite flickering-induced deformations, the topological shape of the coactivity complex remains stable during almost the entire navigation period.

How does the decay of the connections affect the net structure of the flickering complex $F_{\tau }$? As shown on Fig 5A, the numbers of links *N*_2_(*t*) and of the triple connections *N*_3_(*t*) rapidly grow at the onset of the navigation and begin to saturate in about *t*_*s*_ ≈ 4 minutes (i.e., by the time when a typical link had time to make an appearance), reaching their respective asymptotic values in *t*_*a*_ ≈ 7 minutes. To put the size of the resulting flickering complex into a perspective, note that the number of simplexes in a decaying complex $F_{\tau <\infty }$ can never exceed the number of simplexes that would have existed in absence of decay, i.e., in the “perennial” coactivity complex, $F_{\infty }\equiv T$. Thus, the size of the complex at a moment *t*, $F_{\tau }\left(t\right)$, can be characterized by the proportion of simplexes that happened to be actualized at that moment. As illustrated on Fig 5A, these numbers fluctuate around 60% for the second order simplexes and around 40% for the third order simplexes, with the relative variances Δ*N*_2_/*N*_2_ ≈ 12% and Δ*N*_3_/*N*_3_ ≈ 17% respectively. In other words, the perennial coactivity complex $F_{\infty }\left(t\right)$ loses about a half of its size due to the flickering of the simplexes, and fluctuates within about 15% margins from the mean.

To quantify the changes in the complexes’ structure as a function of time, we evaluated the number of two- and three-vertex simplexes that are present at a given moment of time *t*_*i*_, but are missing at a later moment *t*_*j*_, normalized by the size of $F_{\tau }\left(t_{i}\right)$, i.e., $d_{ij}^{\left(k\right)}=N_{k}\left(F_{\tau }\left(t_{i}\right)\backslash F_{\tau }\left(t_{j}\right)\right)/N_{k}\left(F_{\tau }\left(t_{i}\right)\right)$, *k* = 2, 3. As shown on Fig 5B, these numbers, which we refer to, respectively, as the second and third asymmetric distances between $F_{\tau }\left(t_{i}\right)$ and $F_{\tau }\left(t_{j}\right)$, rapidly grow as a function of temporal separation |*t*_*i*_ − *t*_*j*_|. In fact, after approximately the effective decay time $\tau_{e}^{\left(2\right)}$, the difference between $F_{\tau }\left(t_{i}\right)$ and $F_{\tau }\left(t_{j}\right)$ becomes comparable to the sizes of either $F_{\tau }\left(t_{i}\right)$ or $F_{\tau }\left(t_{j}\right)$, which implies that the pool of simplexes in the simplicial complex is replenished at the effective decay timescale. However, the shape of the coactivity complex changes slowly: Fig 5C demonstrates that nearly 100% of the connections are shared at two consecutive moments, i.e., the changes in flickering complex from one moment of time to the next are marginal. Over longer periods, the flickering complex can change significantly. For example, the proportion of simplexes that are present at t_*_ = 9 minutes, when $F_{\tau }$ is particularly inflated, and at other moments, varies around $N_{2}\left(F_{\tau }\left(t_{*}\right)\cap F_{\tau }\left(t\right)\right)\approx 82\%$ for second and $N_{3}\left(F_{\tau }\left(t_{*}\right)\cap F_{\tau }\left(t\right)\right)\approx 64\%$ for the third order simplexes (Fig 5C).

### Topological dynamics

Despite the rapid recycling of the individual simplexes, the large-scale topological characteristics of the flickering complex remain relatively stable. As demonstrated on Fig 5D, after the initial stabilization period of about two minutes (which biologically may be interpreted as the initial learning period), $F_{\tau }$ contains only one zero-dimensional and a single one-dimensional topological loop—as the simulated environment E. Some topological fluctuations appear around t_*_ ≈ 9 minutes, as indicated by an outburst of short-lived spurious loops, most of which last less than a minute. After this period, the first two Betti numbers of $F_{\tau }$ retain their physical values $b_{0}\left(F_{\tau }\right)=b_{1}\left(F_{\tau }\right)=1$ (Fig 5E). Since Zigzag homology theory allows tracing individual loops in $F_{\tau }$ continuously across time, these persistent topological loops can be viewed as ongoing representations of the simply connected environment E and of the physical hole in it. Thus, the coactivity complex $F_{\tau }$ preserves, for the most time, not only its approximate size, but also its topological shape—despite transience at the “microscale”, i.e., at the individual simplex level.

Physiologically, these results indicate that the large-scale topological information significantly outlasts the network’s connections: although in the discussed case about a half of the functional links rewire within a $\tau_{e}^{\left(2\right)}$-period, the topology of the cognitive map encoded by the cell assembly network remain stable. In other words, a transient cell assembly network can encode stable topological characteristics of the ambient space, despite transience of the connections.

### Dependence on the proper decay time *τ*

**Fig 6:**
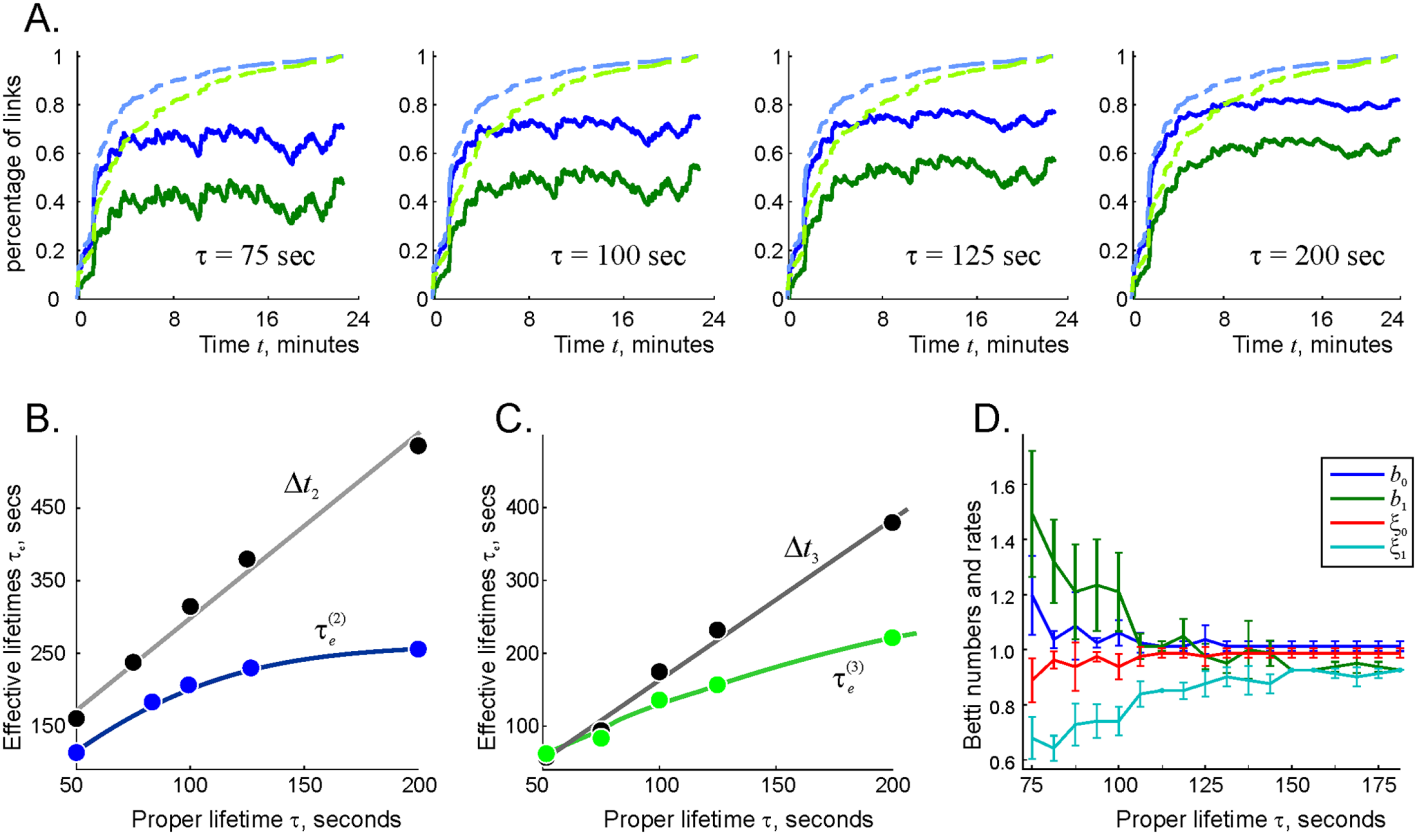
*τ*-dependence of the functional connections and of the topological loops. A: The number of the two-vertex cliques ($N_{2}\left(F_{\tau },t\right)$, green trace) and the number of three-vertex cliques ($N_{3}\left(F_{\tau },t\right)$ blue trace) in the decaying complex, contrasted with the total number of two- and three-vertex connections in the perennial complex across time ($N_{2}\left(F_{\infty },t\right)$ is shown by dashed light-green line and $N_{3}\left(F_{\infty },t\right)$ by light-blue line). The horizontal alignment of panels is used to emphasize increase in the asymptotic values $N_{2,3}\left(F_{\tau },t\gg \tau \right)$. B: The mean lifetimes of the “fluttering” links (i.e., the non-survivor links) in the coactivity complex are about twice longer than the proper link lifetimes, $\tau_{e}^{\left(2\right)}\approx 2\tau$ (blue line). The mean lifetimes of all the links in the coactivity complex (i.e., including the survivor, or “core” links) are about thrice longer than the proper lifetime, $\Delta t_{\varsigma_{2}}\approx 3\tau$ (gray line). C: Same dependences are shown for the triple connections. The effective lifetimes of the short-lived triple connections are approximately equal to proper link lifetime, $\tau_{e}^{\left(3\right)}\approx \tau$. D: The dependence of the Betti numbers of the flickering complex, $b_{k}\left(F_{\tau }\right)$, *k* = 0, 1 and the corresponding percentages of the successful trials $\xi_{k}\left(F_{\tau }\right)$, on the proper decay time demonstrates that the topological fluctuations in $F_{\tau }$ subside as *τ* increases. The results are averaged over 10 place field maps with *N* = 300 randomly scattered place fields. The mean size of the place fields (20 cm) and the mean maximal firing rate of the place cells *f* = 14 Hz is as above.

We investigated the topological stability for a set of proper decay times *τ* ranging from one to five minutes. As one would expect, the number of simplexes in the flickering complex increases with growing *τ*: in the studied map, the number of links raises from about 40% of the maximal value at *τ* = 75 secs to just under 60% for *τ* = 200 secs, whereas the number of the third order connections raises from 60% to about 80% (Fig 6A). The distributions of the effective lifetimes for the short-lived (fluttering) connections retain their exponential shapes (see Suppl. Materials) with the means that are approximately proportional to the proper decay times, $\tau_{e}^{\left(2\right)}\approx 2\tau$ and $\tau_{e}^{\left(3\right)}\approx \tau$ (Fig 6B and 6C). The contribution of the surviving simplexes also steadily grows with *τ* (see Suppl. Materials); as a result, the net average lifetimes, computed for the entire population of simplexes, grow faster $\Delta t_{\varsigma_{2}}\approx 3\tau$ and $\Delta t_{\varsigma_{3}}\approx 2\tau$.

As *τ* increases, the Betti numbers rapidly reduce to their physical values, $b_{0}\left(F_{\tau }\right)=b_{1}\left(F_{\tau }\right)=1$: the lower is the connection decay rate, the smaller are the topological fluctuations generated in the flickering complex (Fig 6D and Suppl. Materials). This is a natural result: the longer the simplexes survive, the closer the topological shape of $F_{\tau }$ is to the topological shape of the environment E. Physiologically, it implies that the lower is the cell assembly decay rate, the more stable is the cognitive map’s topological structure. As shown on Fig 6D, a stabilization of topological barcode is achieved around *τ* ∼ 2 minutes. This value can also be naturally interpreted: for such *τ*, the rat moving at the mean speed of about 25 cm/sec has time to visit most of the environment and reactivate connections in all parts of $F_{\tau }$ before they may decay, which allows the induced coactivity complex to contract the spurious topological loops, to assume and to retain the correct topological shape. Note however, that this is only a qualitative argument since the expected lifetimes of over 63% of links is smaller than *τ* and the lifetimes of 15% of them live longer than 2*τ*.

### Fixed connection lifetimes

**Fig 7:**
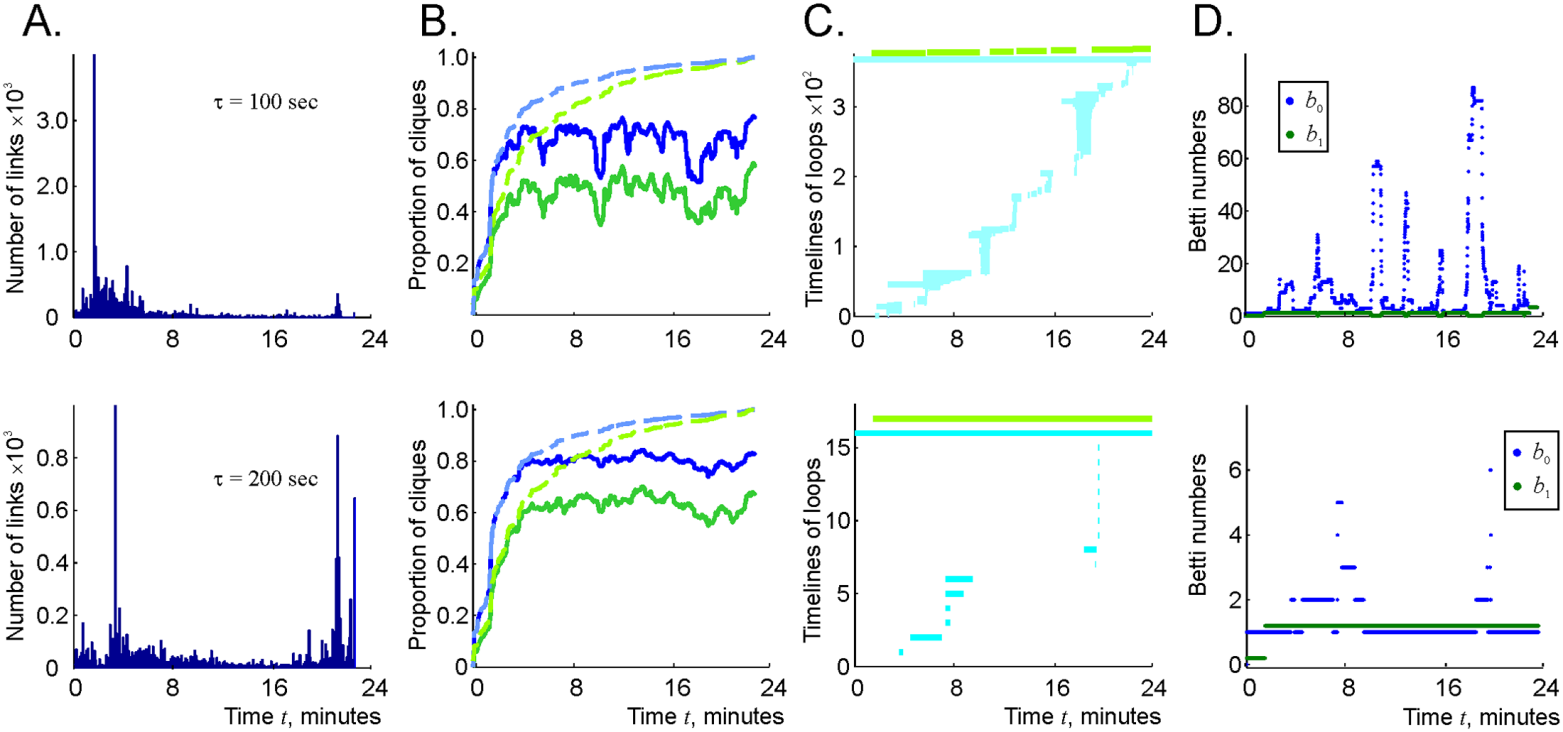
Fixed connection lifetimes lead to topological instabilities. A: The effective timelines of links with the proper decay time of *τ* = 100 secs (top) and *τ* = 200 secs (bottom). The contribution of the links retaining the original, singular proper decay time (*τ*_*e*_ = *τ*) is manifested in the sharp solitary peaks on the left sides of the histograms. The values to the left of that peak are produced by the “boundary effect”: cutting the simulation at *T*_*tot*_ produces timelines shorter than *τ*. B: The distributions of the numbers of two- and three-vertex connections in (green and blue traces) vs. same numbers in the perennial complex $F_{\infty }\left(t\right)$ (dashed lines) indicate that the number of instantiated connections in the case of the singular distribution (2) is higher than in the case of the distribution (1) (see Figs 5A and 6A). As *τ* grows twofold (from 100 to 200 secs) the number of links $N_{2}\left(F_{\tau }^{*}\right)$ grows by 40% and the number of triple connections $N_{3}(F_{\tau }^{*}$) grows by 30%. C: The timelines of 0*D* (light-blue) and 1*D* (light-green) topological loops in the *τ* = 100 secs (top) and in the *τ* = 200 secs (bottom) case. The former produces hundreds of short-lived, spurious loops, while in the latter case there is about a dozen of loops that persist for about 50% of the time. The behavior of the corresponding Betti numbers *b*_0_ (blue) and *b*_1_ (green) is shown on panel D.

To test how these results are affected by the spread of the link lifetimes, we investigated the case in which the lifetimes of all the links are fixed, i.e., the decay probability is defined by the function

$$\begin{array}{c} p\left(t\right)=\{\begin{array}{cc} 1 & \text{if}\;t=\tau \\ 0 & \text{if}\;t\neq \tau , \end{array} \end{array}$$ (2)

while keeping the other parameters of the model unchanged. The results shown on Fig 7A demonstrate that due to the rejuvenation effects, the range of the effective lifetimes widens and becomes qualitatively similar to the histograms induced by the decay distribution (1). As before, there appear two distinct populations of links: the short-lived links whose lifetimes concentrate around the singular proper lifetime *τ*, and the “survivor” links, whose lifetimes approach *T*_*tot*_.

However, the topological structure of the “fixed-lifetime” coactivity complex $F_{\tau }^{*}$ differs dramatically from that of the decaying complex $F_{\tau }$. As shown on Fig 7C, $F_{\tau }^{*}$ contains a large number of short-lived, spurious topological loops even for the values of *τ* that reliably produce physical Betti numbers in the case of the exponentially distributed lifetimes. For example, at *τ* = 100 secs, the zeroth Betti number of $F_{\tau }^{*}$ hovers at the average value of 〈*b*_0_〉 ≈ 40, reaching at times *b*_0_ ∼ 100, with nearly unchanged *b*_1_ = 1, which indicates that $F_{\tau }^{*}$ is split into a few dozens of disconnected, contractible islets.

As the proper decay time increases, the population of survivor links grows and the disconnected pieces of $F_{\tau }^{*}$ begin to pull together: at *τ* = 200 secs, the Betti numbers $b_{k}\left(F_{\tau }^{*}\right)$ retain their physical values most of the time, yielding occasional splashes of topological fluctuations (Fig 7C and 7D).

These differences between the topological properties of $F_{\tau }$ and $F_{\tau }^{*}$ indicate that the tail of the exponential distribution (1), i.e., the statistical presence of long-lasting connections is crucial for producing the correct topology of the flickering complex. Physiologically, this implies that the statistical spread of the connections’ lifetimes plays important role: without it, the dynamical cell assembly network fails to represent the topology of the environment reliably.

### Randomly flickering connections

**Fig 8:**
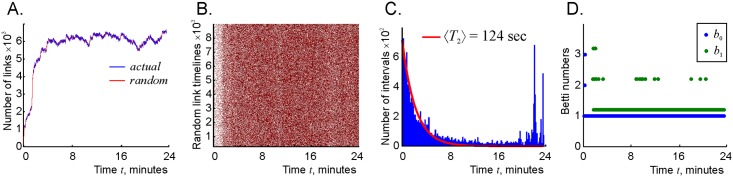
Stochastic complex. A: The number of links in the stochastic coactivity graph $G_{r}\left(t\right)$ (blue trace) is the same as in the decaying coactivity graph $G_{\tau }\left(t\right)$ (red trace). B: The links of the stochastic coactivity graph $G_{r}\left(t\right)$ make instantaneous appearances and disappearances. Compare this chart to the timelines of the links in the decaying coactivity graph $G_{\tau }\left(t\right)$ shown on Fig 3B. C: Histogram of the link’s net lifetimes in the stochastic graph indicates populations of short-lived and survivor links, similarly to the histogram shown on Fig 4G. D: Betti numbers of the stochastic complex stabilize after the initial learning period of about four minutes, indicating the emergence of a stable topological shape of the simplicial complex with stochastically flickering simplexes.

These observations led us to another question: might the topology of the flickering complex be controlled by the shape of the lifetimes’ distribution and the sheer number of links present at a given moment, rather than the specific timing of the links’ appearance and disappearance? To test this hypothesis, we computed the number *N*_2_(*t*) of links in the decaying coactivity graph $G_{\tau }\left(t\right)$ for *τ* = 100 sec at every discrete moment of time *t* (see Methods), and randomly selected the same number of links from the maximal available pool, i.e., from the graph $G_{\infty }\left(t\right)$ that would have formed by that moment without links’ decay (Fig 8A). The collections of links randomly selected at consecutive moments of time can be viewed as instances of a random connectivity graph $G_{r}\left(t\right)$, i.e., as a graph whose links can randomly appear and disappear, in contrast with the decaying links of $G_{\tau }\left(t\right)$ (compare Figs 8B and 3B).

As it turns out, the random and the decaying graphs $G_{r}\left(t\right)$ and $G_{\tau }\left(t\right)$, as well as their respective clique complexes $F_{r}\left(t\right)$ and $F_{\tau }\left(t\right)$ exhibit a number of similarities. First, the histogram of the *net* lifetimes of the links in $G_{r}\left(t\right)$ shown on Fig 8C is bimodal, with an exponential component characterized by the mean 〈*T*_2_〉 = 124 sec, and a component representing a population of surviving connections, similar to the histograms shown on Fig 4G and 4H. Second, the Betti numbers of the random coactivity complex $F_{r}$ converge to the Betti numbers of the environment in about 3 minutes—about as quickly as the Betti numbers of its decaying counterpart $F_{\tau }$ (Fig 8D). However, in contrast with the decaying flickering complex $F_{\tau }$, the random flickering complex $F_{r}$ keeps producing occasional one-dimensional loops over the entire navigational period at a low rate (about 3% of the time, see Suppl. Materials). Thus, according to the model, the topological properties of the map encoded by a network with randomly formed and pruned connections are similar to the properties of a map produced by a network with decaying connections, as long as the net probability of the links’ existence are same. In either case, rapidly rewiring connections do not preclude the appearance of a stable topological map, which once again demonstrates that the latter is a generic phenomenon.

### Compensatory mechanisms

The turnover of memories (encoding new memories, integrating them into the existing frameworks, recycling old memories, consolidating the results, etc.) is based on adapting the synaptic connections in the hippocampal network [47]. In particular, these processes require a balanced contribution of both “learning” and “forgetting” components, i.e., of forming and pruning connections [11, 12]. The imbalances and pathological alterations in the corresponding synaptic mechanisms are observed in many neurodegenerative conditions, e.g., in the Alzheimer’s disease, which is known to affect spatial cognition [48]. However, interpreting the physiological meaning of these alterations is a challenging task, in particular because certain changes in neuronal activity may not be direct consequences of neurodegenerative pathologies. For example, it is believed that neuronal ensembles may increase the spiking rates of the active neurons in order to compensate for the reduced synaptic efficacies [49–55]. Such considerations motivate deep brain stimulation and other treatments that help to improve cognitive performance in animal models of Alzheimer’s diseases and in Alzheimer’s patients, by increasing the electrophysiological activity of hippocampal cells [56, 57].

Previous studies, carried out for the models of perennial cell assembly networks [58], provided a certain theoretical justification for these approaches. It was demonstrated that a place cell ensemble that fails to produce a reliable topological map of the environment due to an insufficient number of active neurons might be forced to produce a correct map by increasing the active place cells’ firing rates. Similarly, the reduction in the firing rates or poor spatial selectivity of spiking may sometimes be compensated by increasing the number of active cells and so forth. Since the current model allows modeling networks with transient connections, we wondered whether it might indicate a theoretical possibility to compensate for the reduced cell assemblies’ lifetimes by altering the place cell spiking parameters.

**Fig 9:**
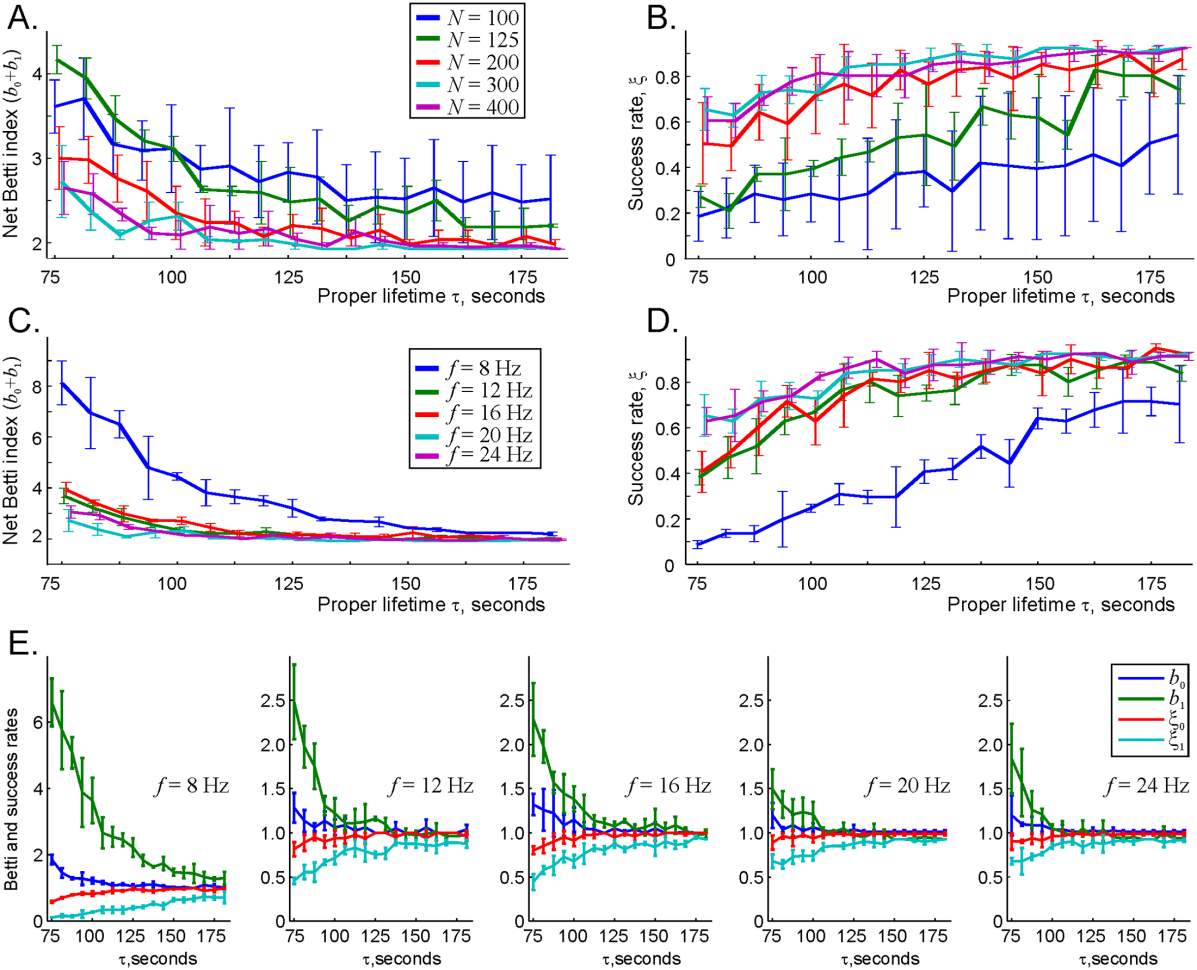
Suppression of the topological fluctuations by increasing neuronal spiking activity. A: As the number of active cells increases, the number of spurious topological loops drops. To compactify the information, we use the sum of the first two Betti numbers, *b* = (*b*_0_ + *b*_1_), which describes the total number of 0*D* and 1*D* loops as a function of decay rate *τ*, computed for several ensemble sizes. As the number of active cells with mean firing rate *f* = 14 Hz increases from *N* = 100 to *N* = 400 cells, the number of loops decrease from 3-4 (indicating at least one spurious loop in 0*D* or in 1*D*) to the physical value $b_{0}\left(E\right)+b_{1}\left(E\right)=2$. B: The proportion of trials—the success rate, *ξ*—in which the coactivity complex produces the correct signature, $b_{k}\left(F_{\tau }\right)=b_{k}\left(E\right)$, as a function of the number of cells, *N*. Larger place cell ensembles tend to represent the topology of the environment more reliably. C: Sum of Betti numbers encoded by an ensemble of *N* = 300 place cells. As the mean ensemble firing rate increases from *f* = 8 to *f* = 24 Hz, the spurious loops die out, i.e., the topological fluctuations in $F_{\tau }$ are suppressed. D: The success rate *ξ*_*k*_ as a function of the decay rate *τ*, computed for *N* = 300 place cells and a set of ensemble mean firing rates. As before, the reliability of the map increases with the ensemble mean rate, for the entire range of the proper decay times. E: Betti numbers $b_{k}\left(F_{\tau }\right)$, *k* = 0, 1, converge to their physical values $b_{k}\left(E\right)$ faster and their respective success rates *ξ*_*k*_ grow more rapidly at higher firing rates.

To that end, we varied the mean firing rate *f* and the number of cells *N* in the simulated place cell ensemble and studied the topological properties of the resulting coactivity complex as a function of the links’ proper half-life, *τ*. The results shown on Fig 9 demonstrate that indeed, increasing neuronal activity helps to suppress topological fluctuations in the flickering coactivity complex for a wide range of the connections’ decay times. Moreover, these changes also increase the proportion of trials in which the place cell ensemble captures the correct signature of the environment (see Suppl. Materials).

Physiologically, these results indicate that recruiting additional active cells and/or boosting place cell firing rates helps to overcome the effect of overly rapid deterioration of the network’s connections, i.e., increasing neuronal activity stabilizes the topological map. In particular, a higher responsiveness of the Betti numbers of the flickering coactivity complex to an increase of the mean firing rate (Fig 9C and 9E) as compared to the number of active place cells (Fig 9A) suggests that targeting the active neurons’ spiking may provide a better strategy for designing clinical stimulation methods.

## Discussion

The formation and disbanding of dynamical place cell assemblies at the short- and intermediate-memory timescales enable rapid processing of the incoming information in the hippocampal network. Although many details of the underlying physiological mechanisms remain unknown, the schematic approach discussed above provides an instrument for exploring how the information provided by the individual cell assemblies may combine into a large-scale spatial memory map and how this process depends on the physiological parameters of neuronal activity. In particular, the model demonstrates that a network with transient connections can successfully capture the topological characteristics of the environment.

Previously, we investigated this effect using an alternative model of transient cell assemblies, in which the connections were constructed by identifying the pool of cells that spike within a certain “coactivity window,” ϖ, and building the coactivity graph $G_{\varpi }$ from the most frequently cofiring pairs of neurons [58]. The accumulation of topological information within each ϖ-period, was then described using persistent homology theory techniques. The results indicate that if ϖ extends over 4-6 minutes or more, the topological fluctuations in the flickering complex are suppressed and the topological shape of $F_{\varpi }$ becomes equivalent to the shape of the environment.

In the current model, enabled by a much more powerful Zigzag persistent homology theory [34–36], we employ an alternative approach, in which the links of the coactivity graph appear instantly following pairwise place cell coactivity events. Thus, in contrast with the model discussed in [58], the current model involves no selection of the “winning” coactivity links, which one might hold responsible for stabilizing the shapes of the flickering coactivity complexes. Nevertheless, this model demonstrates the same effect: the large-scale topological shapes of resulting coactivity complexes stabilize, given that the connections decay sufficiently slowly and have sufficiently broadly distributed lifetimes. The connections’ lifetimes required to achieve such stabilization in the “latency free” model are longer than in the input integration model (*τ* ≈ 100 sec vs. $\tau_{\varpi }\approx 10$ sec), which indicates that physiological networks may integrate spiking information over a certain extended period ϖ and optimize the network’s architecture over this information. However, the fact that stable topological maps can emerge in all these different types of transient networks (including randomly flickering networks) suggests that this is a generic effect that fundamentally may be responsible for the appearance of stable cognitive representations of the environment in the physiological neuronal networks with transient connections. In other words, the emergence of stable topological maps may represent a common “umbrella” phenomenon that can be implemented via different physiological mechanisms.

In all cases, the model reveals three principal timescales of spatial information processing. First, the ongoing information about local spatial connectivity is rapidly processed at the working memory timescale, which physiologically corresponds to rapid forming and disbanding of the dynamical place cell assemblies in the hippocampal network. The large-scale characteristics of space, as described by the instantaneous Betti numbers, unfold at the intermediate memory timescale. At the long-term memory timescale the topological fluctuations average out, yielding stable, qualitative information about the environment. While the former may take place in the hippocampus, the latter two might require involvement of the cortical networks. Thus, the model reaffirms functional importance of the complementary learning systems for processing spatial information at different timescales and at different levels of spatial granularity [47, 59, 60].

## Methods

**Fig 10:**
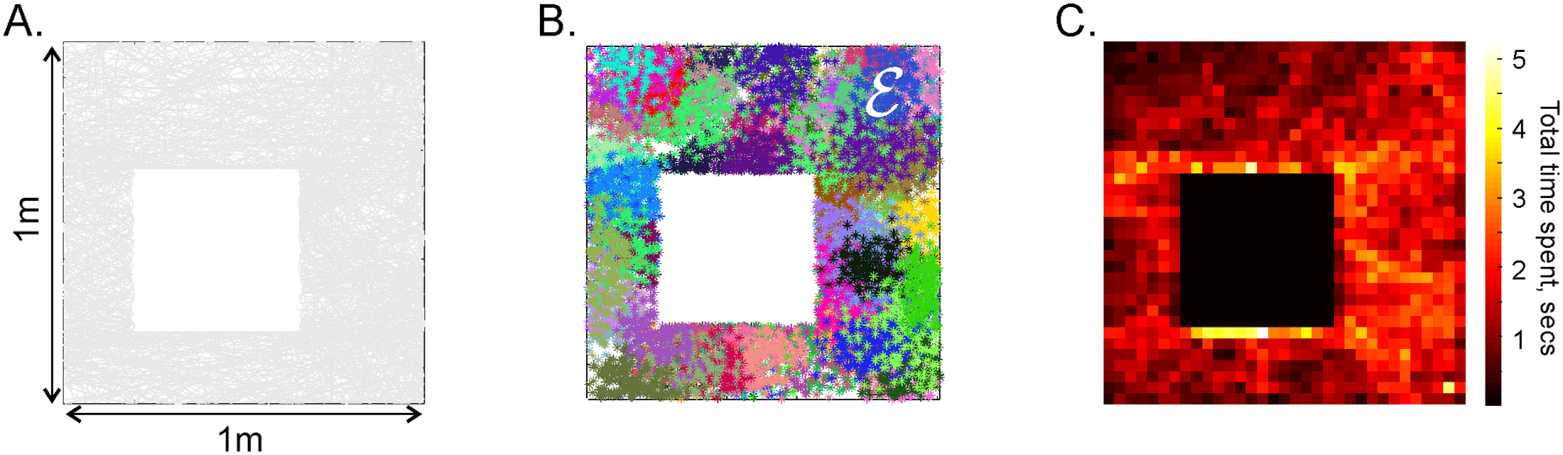
Simulated environment. A: The trajectory covers a small planar arena E uniformly, without artificial circling or other ad hoc favoring of one segment of the environment over another. B: Simulated place field map $M_{E}$. Clusters of dots of a particular color represent spikes produced by the corresponding place cells. C: A 2*D* histogram of the time spent by the animal in different locations—the occupancy map of E.

**Simulated environment** E represents a small (1*m* × 1*m*) square arena with a square hole in the middle, similar to the environments used in electrophysiological experiments [61]. Fig 10 shows the simulated trajectory, the uniform layout of the place fields in the place field map $M_{E}$, and the occupancy map. In [26] we demonstrated that different parts of the environment can be learned independently from one another. Thus, knowing how learning works in smaller domains, one can “map out” learning in larger environments.

**Place cell spiking activity** is modeled as a stationary temporal Poisson process with the maximal firing rate *f*_*c*_ localized at the place field center *r*_*c*_,

$$\begin{array}{c} \lambda_{c}\left(r\right)=f_{c}e^{-\frac{{\left(r-r_{c}\right)}^{2}}{2s_{c}^{2}}} \end{array}$$

where *s*_*c*_ defines the place field’s size [62]. If the place field centers are scattered uniformly over the environment, then an ensemble of *N* place cells, *c* = 1, …, *N*, is defined by 2*N* independent parameters, which we consider as random variables drawn from stationary lognormal distributions with the respective means *f* and *s*. In addition, spiking is modulated by the *θ*-rhythm of the hippocampal extracellular local field oscillations, with the frequency of ∼ 8 Hz [63]. The distributions parameters and the details of the spike simulation algorithms are provided in [25, 26].

### Place cell coactivity

We consider a group of place cells *c*_0_, …, *c*_*k*_ *coactive*, if they produce spikes within two consecutive *θ*-periods [26, 43]. As a result, the time interval [0, *T*_*tot*_] splits into 1/4 sec long time bins that define the discrete time steps *t*_1_, …, *t*_*n*_.

### Simplicial complexes

**Fig 11:**
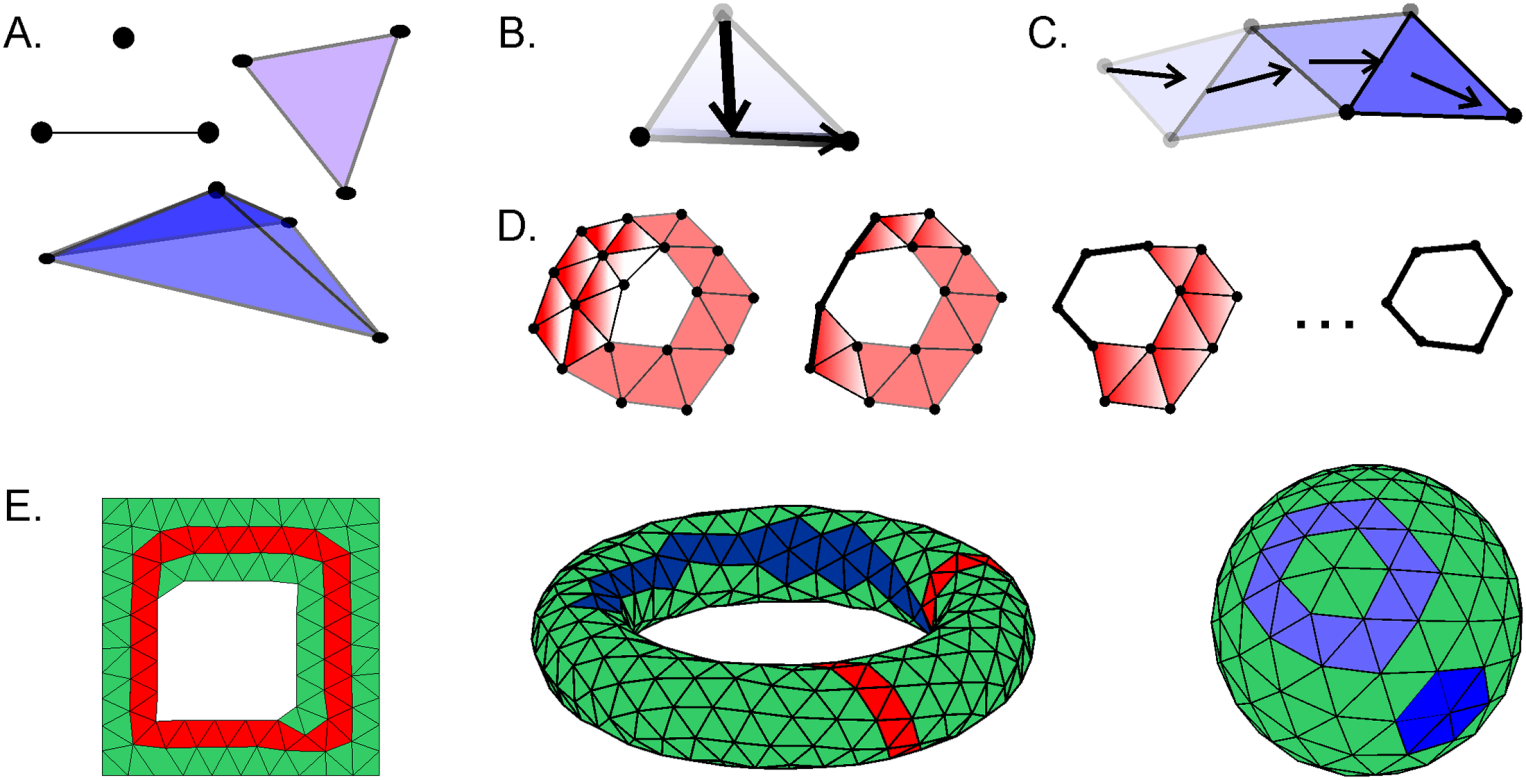
Simplexes and simplicial complexes. A: A zero-dimensional (0*D*) simplex $\sigma_{i}^{0}$ corresponds to a point vertex *v*_*i*_; a one-dimensional (1*D*) simplex $\sigma_{ij}^{1}$—to a link between two vertexes *v*_*i*_ and *v*_*j*_; a two-dimensional (2*D*) simplex $\sigma_{ijk}^{2}$ —to a filled triangle; a three-dimensional (3*D*) simplex $\sigma_{ijkl}^{3}$—to a filled tetrahedron, etc. The *n* vertexes connected by the full set of 1*D* links form cliques, *σ*^*n*^, of the corresponding order. B: A single simplex *σ*^*n*^ is a contractible figure, i.e., it can be collapsed into one of its facets *σ*^*n*−1^, then to a facet of lower dimensionality *σ*^*n*−2^ and eventually to a point *σ*^0^. Shown is a triangle contracting onto its bottom edge and then to the right vertex. C: A linear chain of simplexes bordering each other at a common face is also contractible. The shade of the triangles constituting the chain defines the order in which the triangles can be contracted (the lighter is the triangle, the sooner it contracts) and the arrows indicate the direction of the contractions. D: If a chain of simplexes loops onto itself and encircles a gap in the middle, then it is not contractible. Collapsing the triangles on the sides of such a closed chain produces an equivalent closed loop, which, ultimately, can be reduced to a non-contractible 1*D* loop, but not to a 0*D* vertex (the hole in the middle prevents that). Topologically, the deformed loops are equivalent to one another, i.e., they should be viewed as deformations of the same topological loop. E: Three simplicial complexes: a complex shaped as the environment E (see Fig 2), a toroidal and a spherical complexes (figures obtained using MATLAB mesh generator [64]). Non-contractible topological loops are shown as closed chains of red triangles and contractible loops are shown in shades of blue.

We use simplexes and simplicial complexes to represent combinatorially the topology of the neural activity. An abstract *simplex* of dimensionality *n* is a set containing *n* + 1 elements. A subset of a simplex is called its *face*. A *simplicial complex* is a collection of simplexes closed under the face relation: if a simplex belongs to a simplicial complex, then so do all of its faces (Fig 11).

In the constructions studied in this paper, our simplicial complexes consist of coactive place cells. If all cells {*c*_0_, …, *c*_*k*_} are coactive within a given time window, then so is any subset of them, meaning coactive simplexes form a complex. In fact, because coactivity is defined for a pair of cells, our simplexes are precisely the cliques in the coactivity graph. A simplex {*c*_0_, …, *c*_*k*_} is present if and only if all of its cells are pairwise coactive.

In flickering clique complexes, certain pairwise connections may decay over time, while others appear as time progresses. The effect on the simplicial complex is that some simplexes are removed from the complex, while others are added to it. So we get a sequence of “flickering complexes,” *X*_*i*_, connected by alternating inclusions:

$$\begin{array}{c} X_{1}\subseteq X_{2}\subseteq X_{3}⊇X_{4}\subseteq X_{5}⊇\ldots \end{array}$$

### Cycles, boundaries, and homology

A *k*-dimensional *chain* is a set of *k*-dimensional simplexes (Fig 11) that can be combined with suitable coefficients. If the coefficients form an algebraic field, then the chains form a vector space. Here we use the simplest algebraic field $Z_{2}$, which consists of two Boolean values 0 and 1. A boundary of the simplex is the sum of its one-dimension-lower faces: $\partial_{k}\left\{c_{0},\ldots ,c_{k}\right\}=\sum_{i=0}^{k}\left\{c_{0},\ldots ,c_{i-1},c_{i+1},\ldots ,c_{k}\right\}$. The map extends linearly to the entire simplicial complex, *X*, mapping its *k*-dimensional chains to its (*k* − 1)-dimensional chains. The kernel of this map, i.e., all the chains without a boundary, is the set of *cycles* of the complex, denoted by Z_*k*_(*X*) = ker *∂*_*k*_. The image of ∂_*k*+1_ consists of the *k*-dimensional chains that are *boundaries* of some (*k* + 1)-dimensional chains, denoted by B_*k*_(*X*) = im *∂*_*k*+1_.

Cycles count “*k*-dimensional holes” in the complex. But not all such holes are independent of each other. We consider two cycles equivalent, or *homologous*, if they differ by a boundary. Algebraically, one can verify that boundaries themselves have no boundaries, ∂_*k*_ ∘ ∂_*k*+1_ = 0. In other words, boundaries are cycles. This allows us to take a quotient, H_*k*_(*X*) = Z_*k*_(*X*)/B_*k*_(*X*), called the *k*-dimensional *homology* vector space. By definition, it considers two cycles equivalent, if their difference is a boundary of some (*k* + 1)-dimensional chain. The dimension of this vector space, called the *k*-th Betti number, *β*_*k*_(*X*) = dim H_*k*_(*X*), counts the number of independent holes in the topological space.

### Zigzag persistent homology

Given the sequence of flickering complexes above, we compute homology of each one. Inclusions between complexes induce maps between the homology vector spaces: the homology class of a cycle in the smaller complex maps to the homology class of the same cycle in the larger complex. Accordingly, we get a sequence of homology vector spaces, connected by linear maps:

$$\begin{array}{c} H_{k}\left(X_{1}\right)\to H_{k}\left(X_{2}\right)\to H_{k}\left(X_{3}\right)\leftarrow H_{k}\left(X_{4}\right)\to H_{k}\left(X_{5}\right)\leftarrow \ldots \end{array}$$

This sequence, called *zigzag persistent homology*, generalizes ordinary persistent homology [36], where all the maps between homology groups point in the same direction. It is this generalization to the alternating maps that allows us to handle the flickering complexes.

On the surface, zigzag persistent homology tracks how the Betti numbers of the flickering complexes change. But the maps that connect homology vector spaces provide extra information. It is possible to select a basis for each vector space in this sequence, so that the bases for adjacent vector spaces are compatible [34]. Specifically, we can select a collection of elements ${\left\{z_{i}^{j}\right\}}_{j}$ for each vector space H_*k*_(*X_i_*), such that the non-zero elements form a basis for the homology vector space H_*k*_(*X_i_*)—in other words, they represent a set of independent holes in *X*_*i*_. Furthermore, such collections are compatible in the sense that adjacent basis elements map into each other: if we have a map *f*: H_*k*_(*X_i_*) → H_*k*_(*X*_*i*±1_), then $f\left(z_{i}^{j}\right)=z_{i\pm 1}^{j}$, if $z_{i}^{j}\neq 0$. The experiments in this paper use the algorithm of Carlsson et al. [35] to compute such compatible bases.

It follows that the sequence of homology vector spaces can be decomposed into a barcode, where each bar represents the part of the sequence, where a particular basis element is non-zero. The bars capture when independent holes appear in the flickering complex, how long they persist, and when they eventually disappear. The authors will provide the software used for these computations upon request.

## Supporting information

S1 FigStatistics of the connections’ lifetimes.A: Histograms of the intervals between consecutive births (*b*) and deaths (*d*) of the pairwise ($\Delta t_{\varsigma_{i}}=t_{\varsigma }^{\left(d_{i}\right)}-t_{\varsigma }^{\left(b_{i}\right)}$, left column of panels) and triple ($\Delta t_{\varsigma_{i}^{3}}=t_{\varsigma^{3}}^{\left(d_{i}\right)}-t_{\varsigma^{3}}^{\left(b_{i}\right)}$, right column of panels) connections, for five values of the proper decay times *τ*. The red line outlines the exponentials with proper decay time 1/*τ* and the dark-blue line shows the exponential fit of the histogram with the decay rate 1/*τ*_*e*_, computed for the under 16 minutes long intervals. The exponential fit to the histogram of the effective lifetimes of short-living triple connections ($\Delta t_{\varsigma^{3}}<10$ minutes) is shown by dark-green line on the right panels. The mean lifetime for the entire population of links, Δ*t*_*k*_, is shown at the on each panel.(TIF)

S2 FigLonger decay times suppress topological instabilities.A: Timelines of 0*D* (light-blue) and 1*D* (light-green) topological loops in the flickering coactivity complex, computed for four values of the proper decay time *τ*. B: The corresponding Betti numbers, $b_{0}\left(F_{\tau }\right)$ (blue) and $b_{1}\left(F_{\tau }\right)$ (green).(TIF)

S3 FigTopological properties of the random complex.A. Four tests of the topological behavior of the random complex $F_{r}$ indicate that after initial period of about 3 minutes, this complex produces occasional one-dimensional topological loops in only 3% of the time (success rate *ξ* = 0.97 in all cases). B. The numbers of double and triple connection remains approximately the same from case to case.(TIF)

S4 FigSuppression of topological fluctuations by increasing place cell firing rates.The six consecutive pairs of rows (colors alternate for illustrative purposes) correspond to the ensemble mean firing rate *f* = 12, 14, 16, 18, 20 and 24 Hz. The proper decay time increases along each pair of rows from *τ* = 75 to *τ* = 200 secs, uniformly across the intermediate values. As *τ* increases, the percentage of times (*ξ*) during which the Betti numbers $b_{k}\left(F_{\tau }\right)$, *k* = 0, 1, remain equal to their physical values increases, for all ensemble mean firing rates. The higher is the ensemble mean frequency rate, the smaller are the topological fluctuations across the entire range of *τ*s.(TIF)
