# Supplementary figures and images for: Robust spatial memory maps encoded by networks with transient connections
Source: PLoS Comput Biol. 2018 Sep 18;14(9):e1006433. doi: 10.1371/journal.pcbi.1006433 (PMC6161922; doi:10.1371/journal.pcbi.1006433)

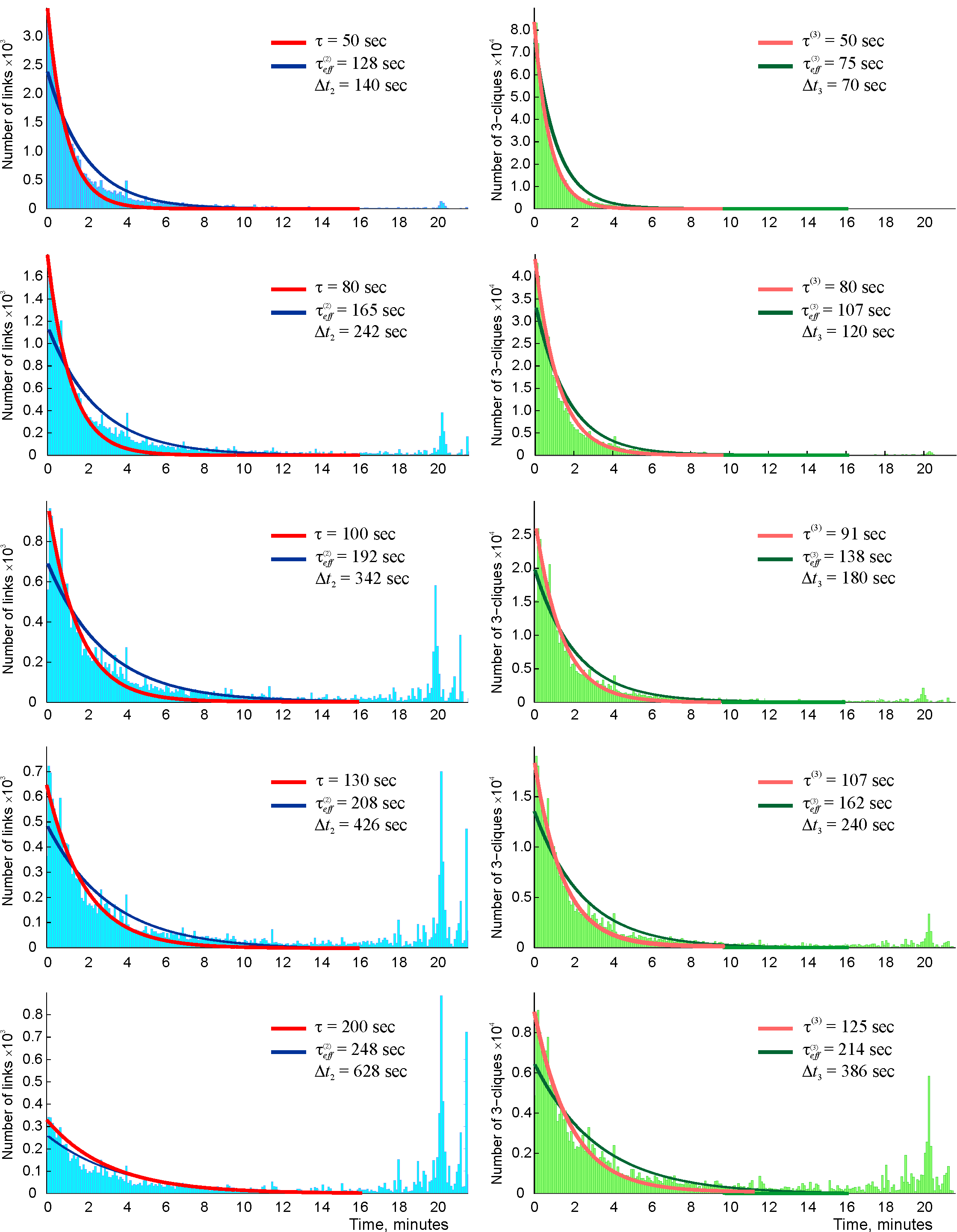

Supplement: S1 Fig — A: Histograms of the intervals between consecutive births (b) and deaths (d) of the pairwise (Δtςi=tς(di)-tς(bi), left column of panels) and triple (Δtςi3=tς3(di)-tς3(bi), right column of panels) connections, for five values of the proper decay times τ. The red line outlines the exponentials with proper decay time 1/τ and the dark-blue line shows the exponential fit of the histogram with the decay rate 1/τe, computed for the under 16 minutes long intervals. The exponential fit to the histogram of the effective lifetimes of short-living triple connections (Δtς3<10 minutes) is shown by dark-green line on the right panels. The mean lifetime for the entire population of links, Δtk, is shown at the on each panel. (TIF) [file pcbi.1006433.s001.tif]

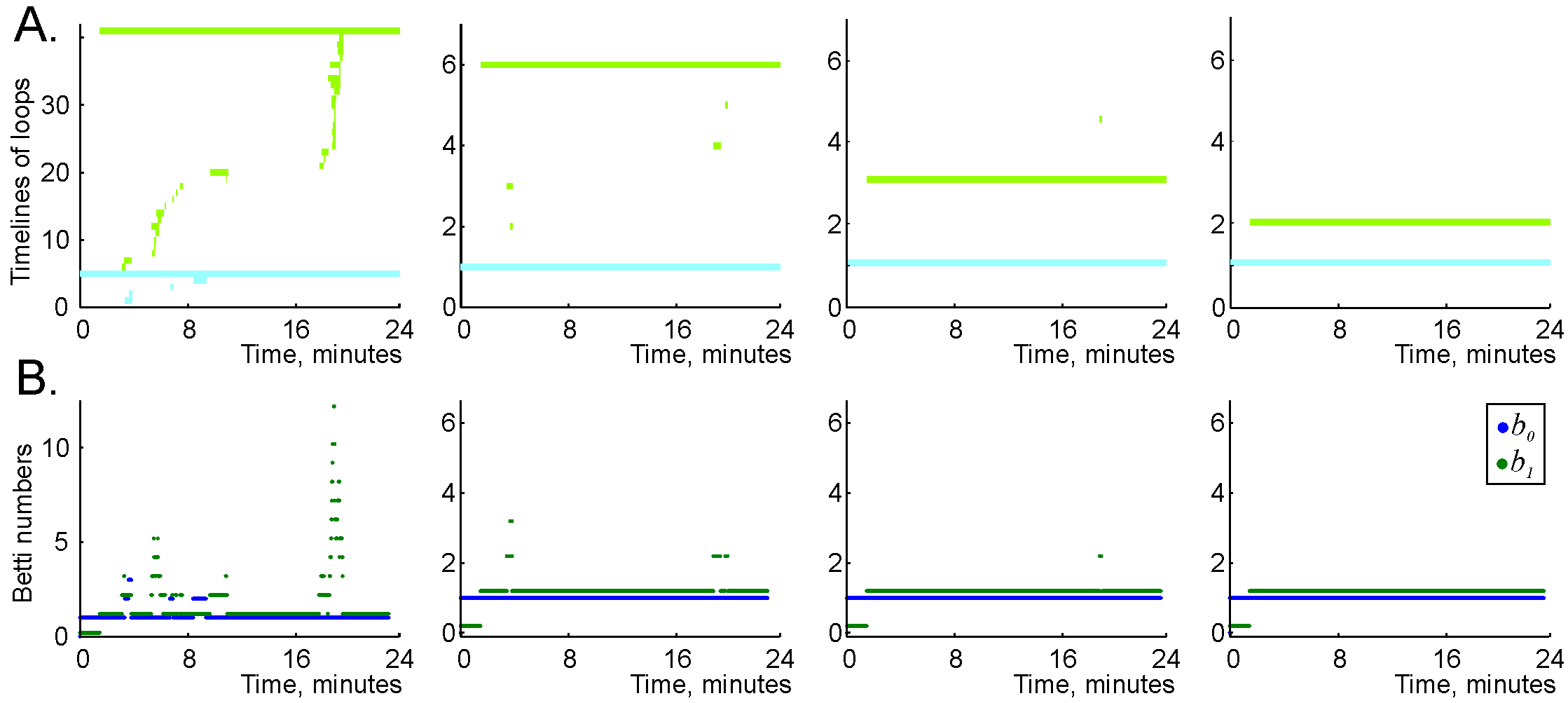

Supplement: S2 Fig — A: Timelines of 0D (light-blue) and 1D (light-green) topological loops in the flickering coactivity complex, computed for four values of the proper decay time τ. B: The corresponding Betti numbers, b0(Fτ) (blue) and b1(Fτ) (green). (TIF) [file pcbi.1006433.s002.tif]

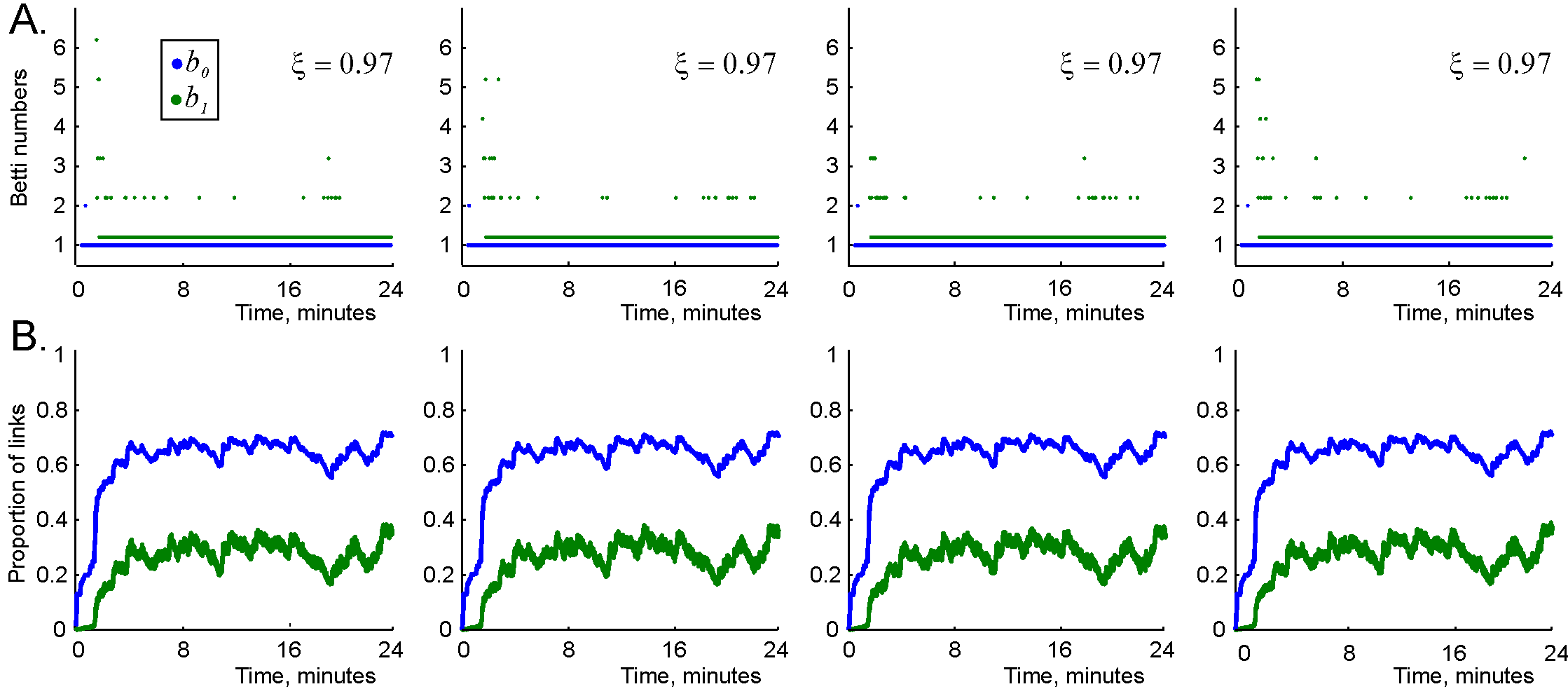

Supplement: S3 Fig — A. Four tests of the topological behavior of the random complex Fr indicate that after initial period of about 3 minutes, this complex produces occasional one-dimensional topological loops in only 3% of the time (success rate ξ = 0.97 in all cases). B. The numbers of double and triple connection remains approximately the same from case to case. (TIF) [file pcbi.1006433.s003.tif]

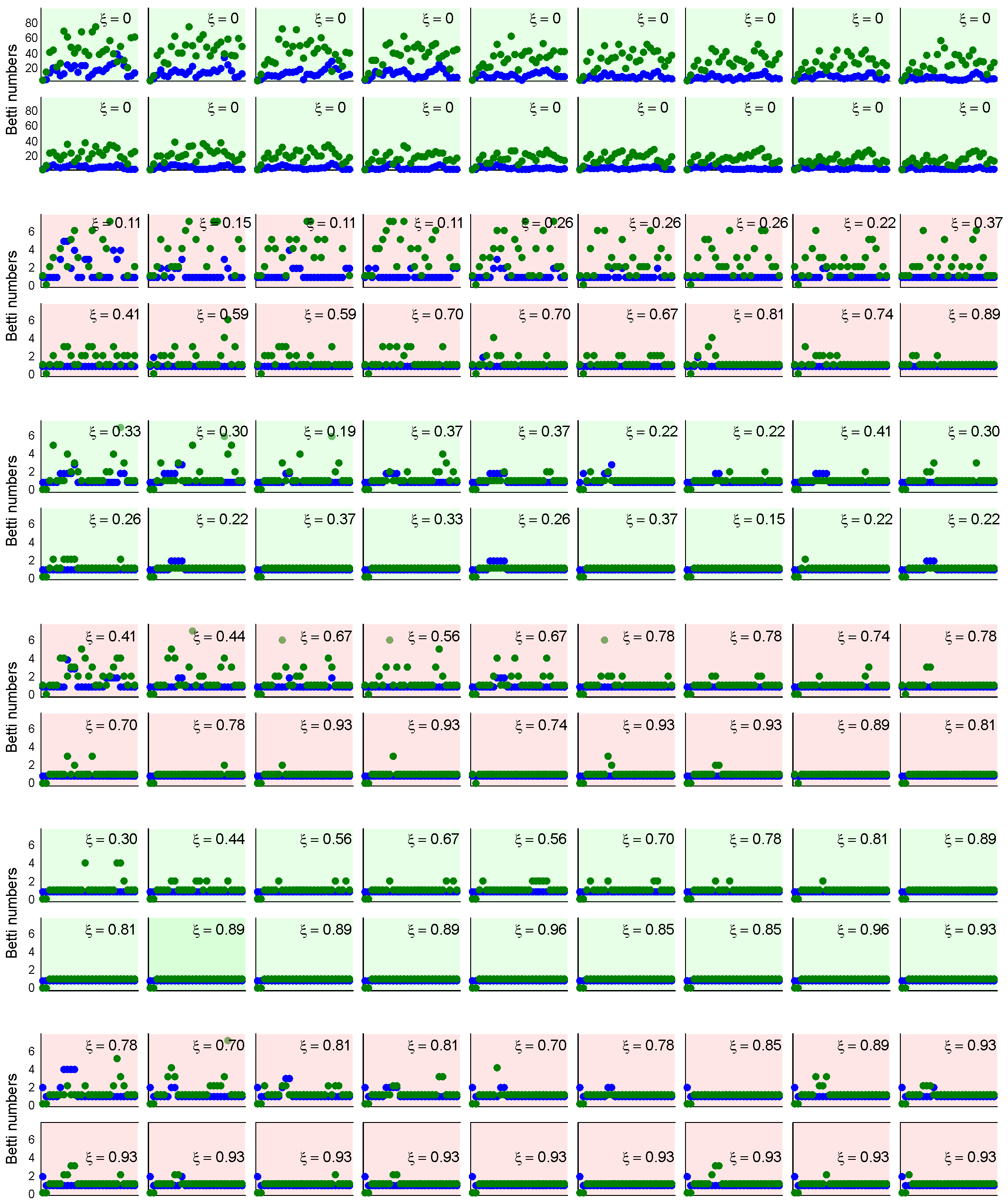

Supplement: S4 Fig — The six consecutive pairs of rows (colors alternate for illustrative purposes) correspond to the ensemble mean firing rate f = 12, 14, 16, 18, 20 and 24 Hz. The proper decay time increases along each pair of rows from τ = 75 to τ = 200 secs, uniformly across the intermediate values. As τ increases, the percentage of times (ξ) during which the Betti numbers bk(Fτ), k = 0, 1, remain equal to their physical values increases, for all ensemble mean firing rates. The higher is the ensemble mean frequency rate, the smaller are the topological fluctuations across the entire range of τs. (TIF) [file pcbi.1006433.s004.tif]
